# Clinical relevance of *Staphylococcus saccharolyticus* detection in human samples: a retrospective cohort study

**DOI:** 10.1007/s15010-024-02334-6

**Published:** 2024-07-04

**Authors:** Ricarda Michels, Cihan Papan, Sébastien Boutin, Farah Alhussein, Sören L. Becker, Dennis Nurjadi, Katharina Last

**Affiliations:** 1 Center for Infectious Diseases, Institute of Medical Microbiology and Hygiene, Saarland University, Homburg, Germany; 2 Institute for Hygiene and Public Health, University Hospital Bonn, Venusberg-Campus 1, Bonn, Germany; 3 Department of Infectious Diseases, Medical Microbiology and Hospital Hygiene, University Hospital Heidelberg, Heidelberg, Germany; 4 Department of Infectious Diseases and Microbiology, University of Lübeck and University Hospital Schleswig-Holstein Campus Lübeck, Lübeck, Germany; 5 Airway Research Center North (ARCN), German center for Lung Research (DZL), Lübeck, Germany; 6 German Center for Infection Research (DZIF), Partner Site Hamburg-Lübeck-Borstel-Riems, Lübeck, Germany

**Keywords:** Coagulase-negative staphylococci, *Staphylococcus saccharolyticus*, Contamination, Infection, Whole-genome sequencing

## Abstract

**Purpose:**

To characterize the clinical relevance of *S. saccharolyticus* and to identify criteria to distinguish between infection and contamination.

**Methods:**

We retrospectively investigated clinical features of patients with *S. saccharolyticus* detection between June 2009 and July 2021. Based on six criteria, infection was considered likely for patients with a score from 3 to 6 points, infection was considered unlikely for patients with a score from 0 to 2 points. We performed group comparison and logistic regression to identify factors than are associated with likely infection. In addition, whole genome sequencing (WGS) of 22 isolates was performed.

**Results:**

Of 93 patients in total, 44 were assigned to the group “infection likely” and 49 to the group “infection unlikely”. Multiple regression analysis revealed “maximum body temperature during hospital stay” to have the strongest predictive effect on likely infection (adjusted odds ratio 4.40, 95% confidence interval 2.07–9.23). WGS revealed two different clades. Compared to isolates from clade A, isolates from clade B were more frequently associated with implanted medical devices (3/10 vs. 9/12, *p* = 0.046) and a shorter time to positivity (TTP) (4.5 vs. 3, *p* = 0.016). Both clades did neither differ significantly in terms of causing a likely infection (clade A 7/10 vs. clade B 5/12, *p* = 0.23) nor in median length of hospital stay (28 vs. 15.5 days, *p* = 0.083) and length of stay at the ICU (21 vs. 3.5 days, *p* = 0.14).

**Conclusion:**

These findings indicate that *S. saccharolyticus* can cause clinically relevant infections. Differentiation between infection and contamination remains challenging.

**Supplementary Information:**

The online version contains supplementary material available at 10.1007/s15010-024-02334-6.

## Introduction

*Staphylococcus* (*S*.) *saccharolyticus* is a rare anaerobic member of the Gram-positive, coagulase-negative staphylococci (CoNS) group, and its pathogenic potential for humans and its clinical impact remain difficult to assess. One of the reasons why CoNS often are misjudged in clinical practice is the difficulty to distinguish between an infection and a contamination [1–4]. CoNS, including *S. saccharolyticus*, constitute a significant part of the human skin microbiome [5, 6]. In combination with its demanding microbiological culturing needs, it can be assumed that *S. saccharolyticus* is often under-detected in the routine microbiological workup of patient samples [7]. Up to date, there remains a dearth of data available on *S*. *saccharolyticus*.

Previously, *S. saccharolyticus* had been primarily associated with prosthetic joint infections and bloodstream infections [8, 9]. In addition, cases of spondylodiscitis [10–12], empyema [13], pneumonia [14] and others have been reported. However, most of these previously published data are limited either because they only pertain to single case reports or case series, and/or they do not include extensive microbiological and genomic characterization.

From a genomic standpoint, *S. saccharolyticus* can be divided into two subclades, A and B. These two subclades differ in subclade-specific genomic islands, e.g., subclade A shows a higher hyaluronidase and urease activity, which might indicate a greater pathogenic potential [8].

The aim of this study was to characterize the clinical relevance of *S. saccharolyticus* detected in human samples, by also taking into account the whole-genome sequencing based characterization, and to identify criteria to distinguish between infection and contamination.

## Materials and methods

### Study design

We conducted a retrospective cohort study at the Saarland University, Homburg, Germany on patients hospitalized between June 2009 and July 2021. The assent of the ethic committee was given (number 147/21). We searched the microbiology laboratory database for all isolates of *S. saccharolyticus* during the defined period. Clinical patient data were extracted from the hospital information system. The following variables were gathered for each patient: sample type in which *S. saccharolyticus* was detected, sex, age, medical history, current diagnosis, overall mortality, length of hospital stay in days, number of positive samples, implanted medical devices, time to positivity, polymicrobial growth, body temperature at admission, maximal body temperature during the hospital stay, body temperature of > 38 °C each two days before and after sampling, heart rate, blood pressure, respiratory rate, C-reactive protein (CRP), leucocyte count, procalcitonin, infection compatible with medical report, antibiotic therapy and antibacterial agent, clinical and laboratory response to therapy, and admission to and length of stay at the intensive care unit (ICU).

### Classification into infection or contamination

We classified each patient case to have an infection or a contamination according to a score consisting of six criteria which were selected based on previous literature [2, 15–18]. One point was awarded for each criterion, with the score having a maximum of six points. The criteria were: *S. saccharolyticus* detected multiples times in different samples of the same patient; absence of other relevant detected pathogens; body temperature > 38 °C two days before and after sampling; CRP > 80 mg/L; a clinical suspicion of infection; and symptom improvement after antibiotic therapy. Patients with a score from 0 to 2 were rated as “infection unlikely/contamination”, patients with a score from 3 to 6 were classified as “infection likely”.

### Whole-genome sequencing (WGS)

We performed WGS to further genetically characterize the isolates. To this end, 22 samples that were available were cultivated on Columbia Blood Agar and incubated under anaerobic conditions for 5 days at 37 °C. Afterwards, we confirmed identification of *S. saccharolyticus* by using matrix-assisted laser desorption/ionization time-of-flight mass spectrometry (MALDI-TOF MS) (Microflex LT and Biotyper 3.1, Bruker Daltonics).

For each isolate, the DNA extraction, library preparation, sequencing on a MiSeq Illumina platform (short-read sequencing, 2 × 300 bp) and post-sequencing procedure were performed as previously described [19]. Raw sequences were controlled for quality using fastp (v0·23·2 with parameters -q = 30 and -l = 45) and assembled with SPAdes 3.15.5 (with the option —careful and—only-assembler) [20, 21]. Draft genomes were curated by removing contigs with a length < 500 bp and/or coverage < 10×. The quality of the final draft was quality-controlled using Quast (v5.0.2) [22]. The complete draft genomes were processed through available databases using Abricate (https://github.com/tseemann/abricate) to identify antimicrobial resistance (NCBI, CARD, ARG-ANNOT, ResFinder, MEGARES databases) and plasmid type (PlasmidFinder database) to identify the Inc type of the plasmid [23, 24]. The genomes were annotated using Prokka v1.14.5 [25] and the core/accessory genome was estimated using Roary [26]. The species identification of each draft genome was done using mash (sub-command screen) by screening each draft genome to a database composed of a representative genome of each species present in the Microbial Genomes resource (https://www.ncbi.nlm.nih.gov/genome/microbes/). As two major clades were discussed in the literature, we compare our draft genomes to published genomes [8, 9]. Each draft genome was aligned to the representative genome reference from the Microbial Genomes resource (CP068029.1, strain 13T0028) using SKA [27]. The alignment was then analyzed with Gubbins 3.2.1 to define hqSNPs distance and phylogenetic relationship [28].

### Antimicrobial susceptibility testing

We carried out antimicrobial susceptibility testing with 19 samples of the total of 22 sequenced strains. We used epsilometry (Liofilchem^®^, Roseto degli Abruzzi, Italy) for determining the Minimum Inhibitory Concentration (MIC) with the following antibiotics: penicillin, piperacillin/tazobactam, meropenem, vancomycin, clindamycin and metronidazole. The results were read out after 48 h of incubation at 35 °C under anaerobic conditions and evaluated according to the EUCAST clinical breakpoints up to Version 11.0.

### Statistical analyses

We used SPSS^®^ (Statistical Product and Service Solutions, IBM^®^, Release 28.0.1.0) for statistical analyses. Groups were compared with χ2 test or Fisher’s exact test for nominally scaled variables, and with Mann-Whitney U test (for non-normally distributed data) and t-test (for normally distributed data) for ordinal or ratio scaled variables. Additionally, a logistic regression was performed to assess potential risk factors associated with a likely infection.

## Results

### Classification into infection or contamination

**Fig. 1:**
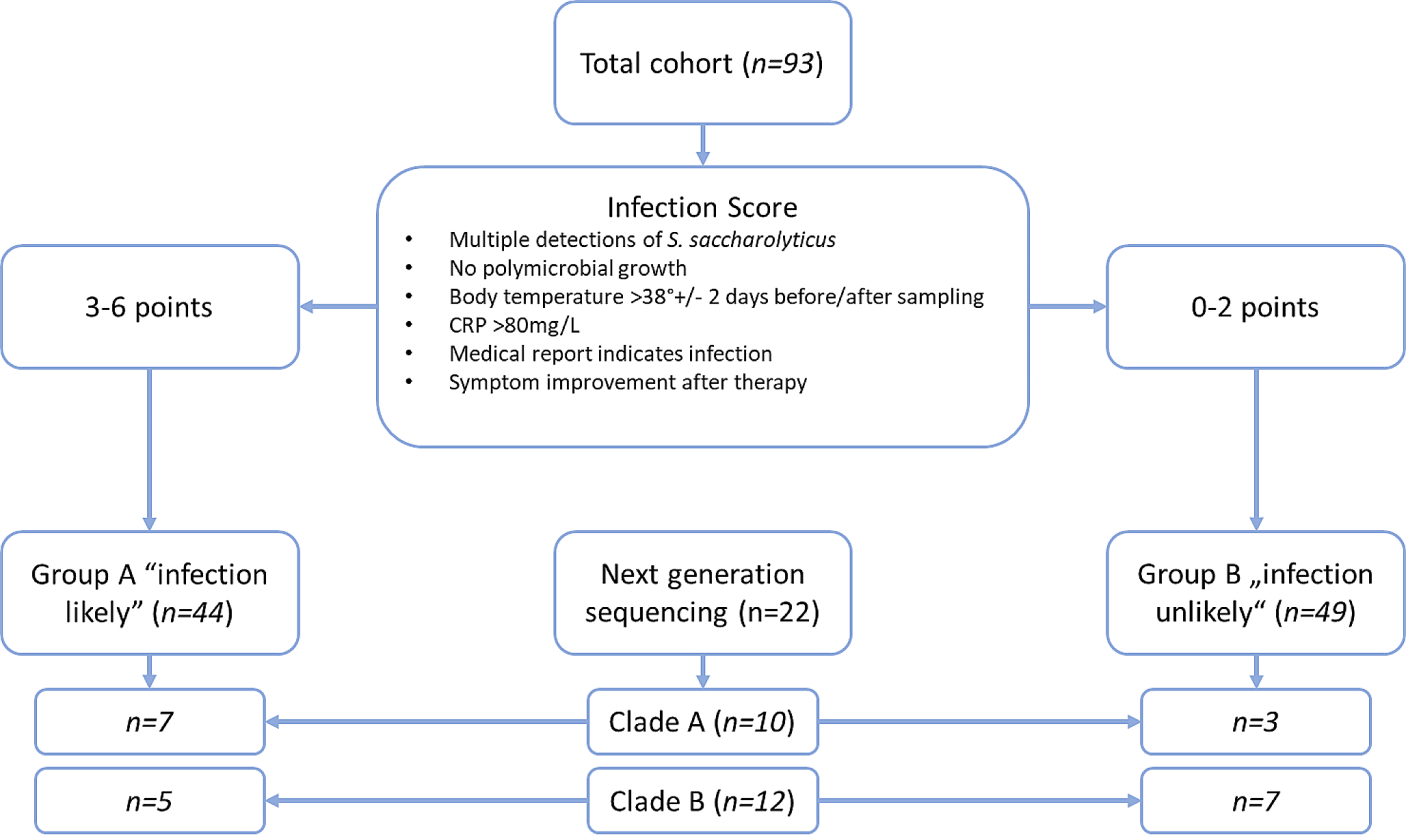
Overview of the cohort and division into the two groups A and B based on the infection score. Additional representation of the sample distribution for next generation sequencing

In total, we identified 93 patients with detection of *S. saccharolyticus* (Fig. 1; Supplementary Table S1). We identified 44 patients with 3–6 points which were classified as group A “infection likely”, whereas 49 patients with 0–2 points were sorted to group B ”infection unlikely”. Table 1 provides an overview of the main characteristics of both groups. The two groups did not differ significantly in sex and median age. There were more men than women in both groups (39/44 [88.6%] in group A and 41/49 [83.7%] in group B, see Table 1). In contrast, other characteristics showed significant differences, for example, whether the type of sample material was a blood culture, median length of ICU stay and antibiotic therapy. Of note, the majority of isolates were detected in blood cultures (57/93), followed by wound swabs (23/93), tissue samples (6/93), skin swabs (3/93), aspirates (2/93) and urine samples (2/93).

**Table 1:** Six criteria included in the score. Overview of all collected features and description of the total cohort; *p*-values refer to the comparison of the two groups; *during the period considered; **TTP = time to positivity (0 = not determined, 1 = sample positive in 0–24 h, 2 = sample positive in 24–48 h, 3 = sample positive in 48–72 h, 4 = sample positive in 72–96 h, 5 = sample positive in 96–120 h, 6 = sample positive in 120–144 h, 7 = sample positive in 144–168 h, 8 = sample positive in 168–192 h

	*Total* (*n* = *93*)	*Infection likely* (*n* = *44*)	*Infection unlikely* (*n* = *49*)	*p-value*
*Criteria included in the score*				
Multiple detections of *S. saccharolyticus* (n/N, %)	6/93 (6.5)	5/44 (11.4)	1/49 (2)	0.097
absence of other relevant detected pathogens^a^ (n/N, %)	60/90 (66.7)	33/44 (75.0)	27/46 (58.7)	1.121
Body temperature > 38° +/- 2 days before/after sampling^b^ (n/N, %)	36/71 (50.7)	33/41 (80.5)	3/30 (10.0)	< 0.001
CRP > 80mg/L^c^ (n/N, %)	44/79 (55.7)	31/44 (70.5)	13/35 (37.1)	0.006
Medical report indicates infection^d^ (n/N, %)	47/72 (65.3)	41/43 (95.3)	6/29 (20.7)	< 0.001
Symptom improvement after therapy^e^ (n/N, %)	33/46 (71.7)	28/39 (71.8)	5/7 (71.4)	1
*All collected features*				
Male sex (n/N, %)	80/93 (86.0)	39/44 (88.6)	41/49 (83.7)	0.56
Age (years) (median, IQR)	56.2 (44.5–66.8)	57.7 (45.7–69.4)	55.7 (42.7–64.2)	0.29
Blood culture as sample (n/N, %)	57/93 (61.3)	37/44 (84.1)	20/49 (40.8)	< 0.001
In-house mortality* (n/N, %)	5/91 (5.5)	4/44 (9.1)	1/47 (2.1)	0.18
Comorbidities (n/N, %)	76/90 (84.4)	39/44 (88.6)	37/46 (80.4)	0.39
Length of hospital stay (days)^1^ (median, IQR)	15 (7–34)	29 (13–41)	10 (5–17)	< 0.001
Implanted medical devices (n, N %)	42/86 (48.8)	23/44 (52.3)	19/42 (45.2)	0.53
TTP **^2^ (median; IQR)	5 (3–6)	5 (4–6)	4 (3–6)	0.10
Temperature at admission^3^ (°C) (median, IQR)	36.6 (36–37.2)	36.55 (35.7–37.5)	36.6 (36–37.2)	0.99
Max. temperature (°C)^4^ (median, IQR)	38.5 (37.6–39.2)	39.1 (38.5–40)	37.5 (37.2–38)	< 0.001
Heart rate (bpm)^5^ (median, IQR)	90 (80–100)	93 (80–104)	90 (80–96)	0.13
Blood pressure, systolic (mmHg)^4^ (median, IQR)	125 (110–140)	125 (110–139)	130 (110–145)	0.15
Blood pressure, diastolic (mmHg)^4^ (median, IQR)	70 (59–80)	70 (50–80)	71 (67–80)	0.15
C-reactive protein (mg/L)^6^ (median, IQR)	98.05 (35.4–193.9)	142.6 (65.4–230.2)	62.55 (8.1–154)	0.0044
Leukocyte count (10^9^/L)^6^ (median, IQR)	9.85 (7.7–13.7)	11.45 (8.4–15.7)	9.2 (6.8–12)	0.07
Procalcitonin (ng/mL)^7^ (median, IQR)	0.9 (0.3–3.4)	0.95 (0.4–1.6)	0.43 (0.1–3.56)	0.58
Antibiotic therapy (n/N, %)	72/82 (87.8)	43/44 (97.7)	29/38 (76.3)	0.0047
Improvement after therapy (n/N, %)	34/50 (68)	24/38 (63.2)	10/12 (83.3)	0.29
ICU stay (n/N, %)	51/81 (63)	34/43 (79.1)	17/38 (44.7)	0.0024
Length of ICU stay (days)^8^ (median, IQR)	7 (2–23)	13 (4–34)	3 (1–5)	0.0024

^a^missing data in 3 cases; ^b^missing data in 22 cases; ^c^missing data in 14 cases; ^d^missing data in 21 cases; ^e^missing data in 47 cases; ^1^missing data in 4 cases; ^2^missing data in 37 cases; ^3^missing data in 35 cases; ^4^missing data in 24 cases; ^5^missing data in 33 cases; ^6^missing data in 13 cases; ^7^missing data in 70 cases; ^8^missing data in 43 cases

Univariate regression identified four significant features, namely “length of hospital stay” (*p* = 0.004), “maximal body temperature during the hospital stay” (*p* < 0.001), “antibiotic therapy” (*p* = 0.017) and “stay at ICU” (*p* = 0.002) (Table 2). In multiple regression analysis, “maximal body temperature during hospital stay” had the strongest predictive effect with an adjusted odds ratio of 4.40 (95% confidence interval, CI, 2.07–9.23; *p* < 0.001) (Table 2).

**Table 2:** Results of the multivariate regression analysis with the four significant features of the univariate analysis; Nagelkerke’s R2 0.527 OR: odds ratio; CI: confidence interval; aOR: adjusted odds ratio

	OR	95% CI	*p*-value	aOR	95% CI	*p*-value
Length of hospital stay	1.04	1.01–1.07	0.004	-	-	-
Maximal temperature	4.85	2.27–10.33	< 0.001	4.40	2.07–9.23	< 0.001
Antibiotic therapy	0.075	0.009–0.624	0.017	0.02	0.0–1.79	0.088
ICU stay	0.214	0.08–0.57	0.002	-	-	-

### Whole-genome sequencing (WGS)

**Fig. 2:**
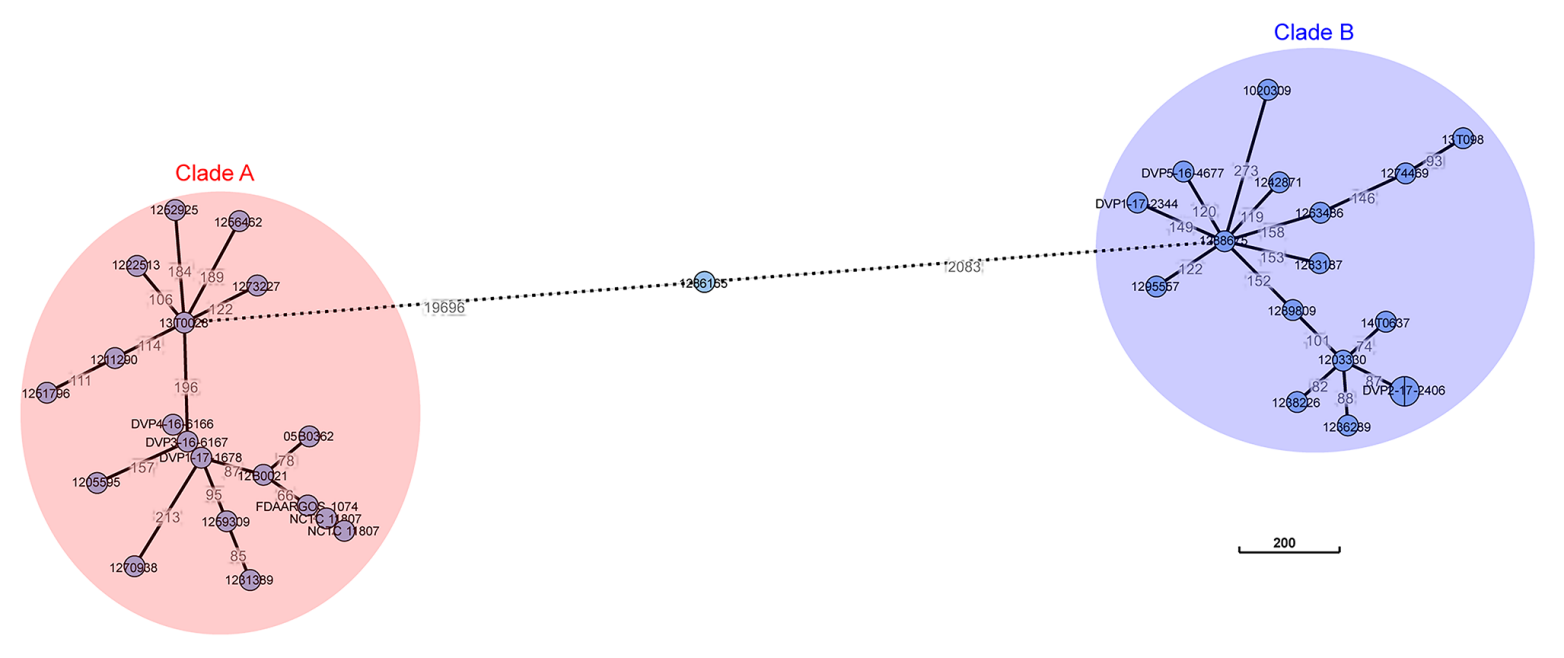
Minimum spanning tree representing the phylogeny of the *Staphylococcus saccharolyticus* population, based on core-genome comparison. Available *S. saccharolyticus* genomes were compared, including publicly available sequenced genomes and strain from our study (22 strains)

WGS was performed on 12 and 10 samples from each group, respectively. Remaining patient samples were not recoverable and isolates were unavailable for sequencing. WGS revealed two clades, clade A and clade B as well as one outlier in between the two clades for which the SNPs distance is too high to be included in the clade B (Fig. 2). Comparison of both clades revealed significant clade-specific features (Table 3). Infection was likely among isolates from clade A in 7/10 cases, vs. 5/12 among clade B. Data on infections per clade are shown in the Appendix (Supplementary Table S2). Furthermore, in our study, there were more patients with medical devices in clade B compared to clade A (9/12 vs. 3/10, *p* = 0.046). However, these patients did not always necessarily have an infection (i.e. 4/9 in clade B with medical device had an infection, while 2/3 patients from clade A with a medical device had an infection). The median time to positivity (TTP) in clade A and B were 4.5 and 3, respectively, (*p* = 0.016). We found the median length of hospital stay and of stay at the ICU to be higher in clade A (28 days and 21 days) than in B (15.5 and 3.5 days, respectively), although not reaching statistical significance. Similarly, median heart rate was higher in clade A than in B (A: 90.5; B: 87), as were the median systolic and diastolic blood pressure (A: 124.5/70.5; B: 116/62.5) and the median CRP (A: 113.15; B: 96.7).

**Table 3:** Comparison of clade A and B; *overall mortality: during the period considered; **TTP = time to positivity (0 = not determined, 1 = sample positive in 0–24 h, 2 = sample positive in 24–48 h, 3 = sample positive in 48–72 h, 4 = sample positive in 72–96 h, 5 = sample positive in 96–120 h, 6 = sample positive in 120–144 h, 7 = sample positive in 144–168 h, 8 = sample positive in 168–192 h

	Clade A (*n* = 10)	Clade B (*n* = 12)	*p*-value
Male sex (n/N, %)	8/10 (80)	11/12 (91.7)	0.57
Age (years) (median, IQR)	61.6 (54.4–71.5)	54.5 (46.6–70.7)	0.47
Blood culture as sample (n/N, %)	10/10 (100)	12/12 (100)	1.00
In-house mortality* (n/N, %)	1/10 (10)	2/12 (16.7)	0.57
Comorbidities (n/N, %)	10/10 (100)	12/12 (100)	1.00
Length of hospital stay (days) (median, IQR)	28 (12–50)	15.5 (7–29)	0.083
Implanted medical devices (n/N, %)	3/10 (30)	9/12 (75)	**0.046**
TTP**^1^ (median, IQR)	4.5 (4–6)	3 (3–4)	**0.016**
Temperature at admission (°C)^2^ (median, IQR)	36.7 (35.7–36.9)	36.7 (36.5–38.5)	0.59
Maximal temperature during hospital stay (°C)^3^ (median, IQR)	38.6 (37.9–40.1)	38.5 (37.5–39.4)	0.59
Heart rate (bpm)^4^ (median, IQR)	90.5 (72–130)	87 (81–93)	0.37
Blood pressure, systolic (mmHg)^5^ (median, IQR)	124.5 (110–140)	116 (98–125)	0.19
Blood pressure, diastolic (mmHg)^5^ (median, IQR)	70.5 (50–81)	62.5 (56–79)	0.89
C-reactive protein (mg/L)^6^ (median, IQR)	113.15 (31.7–253.6)	96.7 (13.8–171.8)	0.61
Leukocyte count (10^9^/L)^6^ (median, IQR)	10 (13 − 7.7)	9.7 (4.6–20)	0.42
Procalcitonin (ng/mL)^7^ (median, IQR)	0.9 (-)	1.15 (-)	0.58
Antibiotic therapy (n/N, %)	8/10 (80)	8/10 (80)	1.00
Improved laboratory conditions after therapy (n/N, %)	2/6 (33.3)	3/5 (60)	0.57
ICU stay (n/N, %)	5/10 (50)	6/9 (66.7)	0.65
Length at ICU (days)^8^ (median, IQR)	21 (11.5–36.5)	3.5 (1–18)	0.14
Infection likely (n/N, %)	7/10 (70)	5/12 (41.7)	0.23

^1^missing data in 2 cases; ^2^missing data in 7 cases; ^3^missing data in 5 cases; ^4^missing data in 8 cases; ^5^missing data in 4 cases; ^6^missing data in 1 case; ^7^missing data in 17 cases; ^8^missing data in 11 cases

**Fig. 3:**
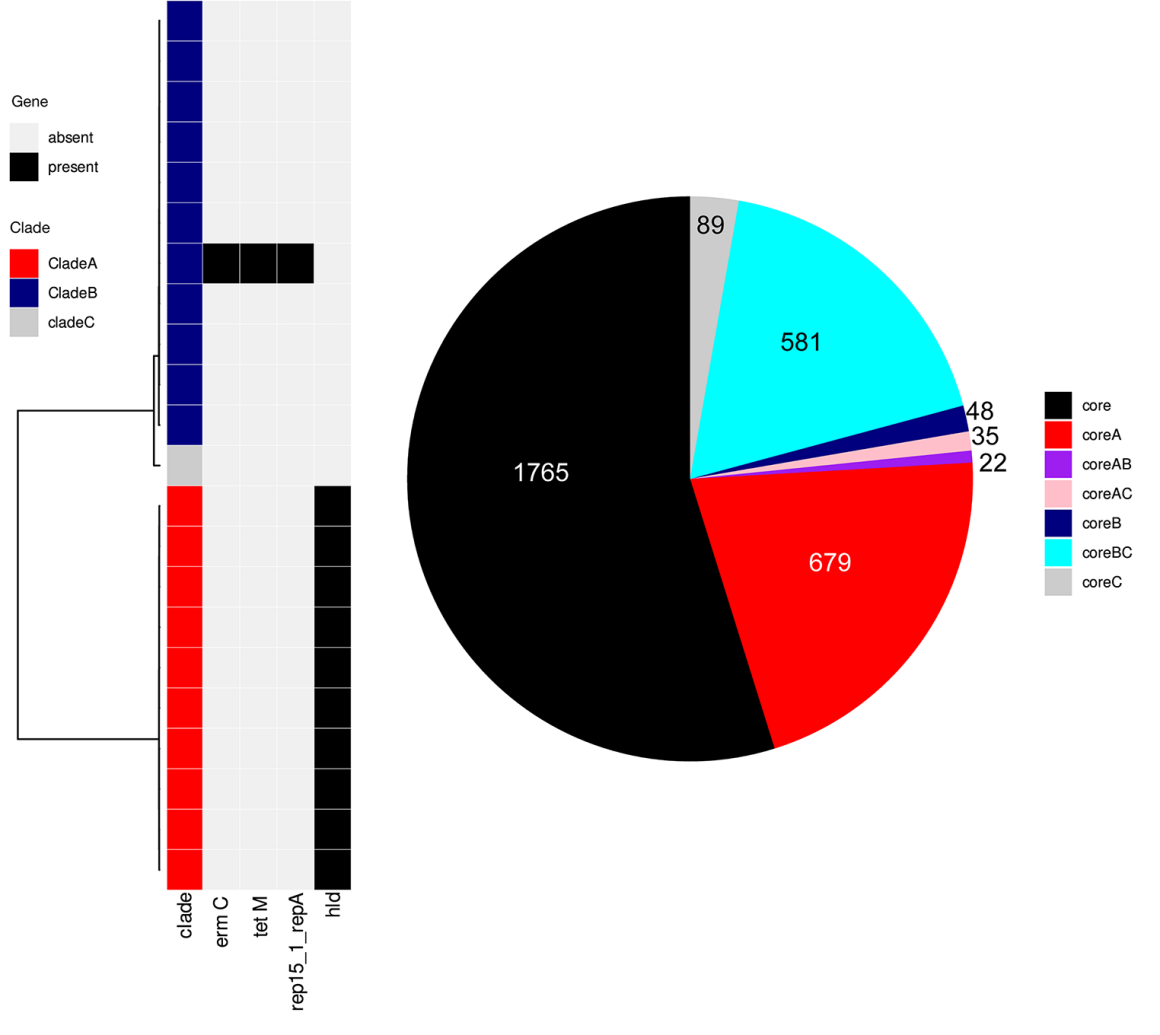
Core genome distribution between the clade A and B. The isolates are ordered based on a phylogenetic tree obtained using Gubbins. The antimicrobial resistance genes are determined using Abricate, the plasmid Inc type using Plasmidfinder and the virulence gene based on the VFDB database. The core/accessory genomes were evaluated using Roary

The core genomes, considered as genes clustering with a 95% identity and present in the whole population (i.e. prevalence of 100%), cover 1765 genes and 2,361,246 bp. For the 100% identity analysis, we used 100% identity and 100% coverage thresholds. 679 genes were only present in clade (A) The isolates from clade A all carried the delta-hemolysin gene *hld*. Only 48 genes were specific to clade B only, but 581 genes were absent in clade A but present in clade B and clade C. However, the core genome is quite small when a threshold of 100% identity is used. Only 62 predicted proteins are shared between clade A and (B) Overall, clade A present a higher protein-encoding content (average of 2651.4 ORFs) compared to clade B (average of 2606.1 ORFs) and a higher conserved core proteome (2115 ORFS predicted in the core-proteome vs. 1809 ORFS predicted in the core-proteome) indicating a higher stability and probably a lower genome decay (Fig. 3).

### Antimicrobial susceptibility testing

Out of 22 sequenced strains, antimicrobial susceptibility testing results of 19 strains were available, while in three cases, the isolate was not culturable for susceptibility testing. The results showed phenotypic susceptibility to penicillin (19 out of 19 isolates), vancomycin (19/19), meropenem (13/13), piperacillin/tazobactam (12/12), and clindamycin (15/16) and showed resistance against metronidazole (12/12 isolates) and clindamycin (1/16 isolates). In only one strain of clade B the *ermC* and *tet* gene were present.

## Discussion

In this retrospective cohort study on patients with *S. saccharolyticus* detection, we found that patients with a likely infection had more often pathogen detection in blood culture(s), a longer length of hospital stay, a higher maximal body temperature during the hospital stay, higher CRP levels, were more likely to be treated with antibiotics, were more likely to be treated at an ICU, and had a longer stay at an ICU. This could point to the pathogenic potential of *S. saccharolyticus* for humans to cause severe illness in systemic infections.

In most cases of *S. saccharolyticus* detection in blood cultures, TTP was longer than 4 days, which, along with the additional 24–48 h needed for antimicrobial susceptibility testing, might have led to suboptimal pathogen-targeted medical treatment. The slow growth of *S. saccharolyticus* remains an issue in diagnosis. The frequently used short TTP as an indicator for infection does not seem to be applicable for differentiation between infection or contamination of *S. saccharolyticus*.

Our regression analysis indicated an association between the maximal body temperature during the hospital stay and likely infection. However, the potential use of this variable as a predictor for infections caused by *S. saccharolyticus* remains questionable, since this parameter can only be assessed retrospectively after the hospital stay. Because the distinction between infection and contamination remains difficult based on clinical parameters alone, genetic markers may be helpful. For example, knowledge about the clade may be used as a predictive marker for disease severity.

Next-generation sequencing revealed two clades of *S. saccharolyticus*, A and B, in line with published data [8]. The distribution of our isolates in these two clades is relatively even. The findings of Brüggemann and colleagues indicates a possibly higher pathogenicity of clade A, which exhibited higher activities of two virulence factors, urease and hyaluronidase [8]. Our clinical data supports this observation: a higher proportion of isolates from clade A were found in patients with a probable infection in our cohort (isolates of clade A: 7/10 (70%) in group “infection likely” versus isolates of clade B: 5/12 (41.6%) in group “infection likely”). In addition, patients with clade A isolates stayed longer at the hospital, and showed higher median heart rate, blood pressure, and CRP levels.

Surprisingly, the presence of a medical device does not seem to have a relevant impact on the occurrence of an infection with *S. saccharolyticus*, unlike with other CoNS. A literature review of cases with *S. saccharolyticus* yielded that 17 of 56 previously published cases were foreign-body related infections [8–10, 29–31]. In our study, there were significantly more patients in clade B with implanted medical devices (9/12 (75%)) than in clade A (3/10 (30%)). However, the infection rate among patients with medical devices was not higher for clade B (4/9) compared to clade A (2/3). Due to the small event number and the lack of more detailed information, it is difficult to compare with previous literature, which had described clade B to be more prevalent in hip joint prosthetic infections [9].

TTP was shorter in clade B, which suggests, contrary to previous assumptions of higher pathogenicity of clade A, that the pathogenicity of this clade B might have been underestimated. The shorter TTP in clade B and a presumable faster growth as a result, could be a potential fitness advantage. Especially considering the lower protein-encoding content and lower conserved core proteome of clade B, the ongoing adaption to a new niche is probable and faster growth could be beneficial [8, 9]. The association between clades and virulence may provide a potential explanation for our findings regarding infection vs. contamination, which warrants further scrutiny.

Other scores to differentiate between infection and contamination were already proposed, for example by Hitzenbichler and colleagues [2] and Asai and colleagues [32]. Both studies aimed to evaluate the clinical relevance of CoNS other than *Staphylococcus epidermidis*. The scores are similar and consider infection likely if, for example, several blood cultures are positive or if there is an improvement in symptoms after therapy. Although *S. saccharolyticus* does not seem to play a particularly important role compared to other CoNS, our score is more differentiated, as it only compares positive *S. saccharolyticus* samples with each other. This allows a better evaluation than in a broader score or in comparison with more relevant CoNS, such as *S. haemolyticus*.

Our study has limitations. The proposed scoring system to distinguish between contamination and infection requires further external validation, i.e. ideally in another cohort within another setting and time, and also taking into account different weightings of individual criteria, which we did not investigate. Since we analyzed only detections of *S. saccharolyticus*, our score might overrate the importance of positive samples with *S. saccharolyticus*. Future validations could also extend to other members of the heterogeneous CoNS group. Another limitation is the inter-rater subjectivity of some variables such as “medical report indicates infection” and “symptom improvement after therapy”. Physicians have probably used similar parameters, such as CRP or temperature, to diagnose infection. This makes retrospective evaluation difficult. Prospective data collection by using standardized case report forms would help to address this limitation in future studies, albeit some imprecision with regard to clinical suspicion of infection might persist. Another limitation is missing data due to the retrospective character of the study, as well as the relatively low number of analyzed isolates of *S. saccharolyticus*, including those available for NGS and/or susceptibility testing, partly due to isolates not being culturable for susceptibility testing despite all efforts. Several parameters could not be ascertained, because the data were not documented or no longer available.

Furthermore, there is a certain degree of incorporation bias since the infection score that we generated on the basis of six criteria contained aspects that can be regarded as part of the clinically accepted reference standard, e.g., a CRP above a certain threshold. This is a common limitation in situations where a clear diagnostic gold standard is missing [33].

Here we describe the largest cohort of *S. saccharolyticus* samples to date. Of 93 patients, 44 (47.3%) were classified with likely infection. This corresponds to almost half of all detected samples and indicates a higher clinical relevance than initially assumed and previously reported [2]. Parameters like TTP, often used to differentiate between infection and contamination, seem to have less of a predictive meaning for *S. saccharolyticus*. This might contribute to misidentification as contamination.

In summary, our findings suggest that the role *S. saccharolyticus* as a clinically relevant pathogen may be underestimated. Further research is necessary to investigate the virulence of clade A and to identify additional predictive factors that can help to distinguish between contamination and infection. This will enable more accurate diagnosis and management of *S. saccharolyticus* infections to improve patient outcomes.

## Electronic supplementary material

Below is the link to the electronic supplementary material.

Supplementary Material 1

Supplementary Material 2

## Data Availability

All data supporting the findings of this study are available within the paper and its Supplementary Information. Genome sequences of the isolates investigated are available at the NCBI GenBank under project number PRJNA1070429.
