# Supplementary material for: Clinical relevance of Staphylococcus saccharolyticus detection in human samples: a retrospective cohort study
Source: Infection. 2024 Jul 4;53(1):145–53. doi: 10.1007/s15010-024-02334-6 (PMC11825615; doi:10.1007/s15010-024-02334-6)
Supplement: Supplementary file 1 — Supplementary Material 1 [file 15010_2024_2334_MOESM1_ESM.docx]

**Online Supplement to: Michels et al. Clinical significance of *Staphylococcus saccharolyticus*: a retrospective cohort study**

**Supplementary Table S1.** Full data set of all included patients (n= 93)

| A )IdentNr B) Sex *(0=male, 1=female)* C) age *(in years)* D) blood culture as sample *(0=no, 1=yes)* E) comorbidities *(0=no, 1=yes)* F) in-house mortality *(0=no, 1=yes)* G) length of hospital stay *(in days)* H) multiple detection of S. saccharolyticus *(0=no, 1=yes)* I) implanted medical devices *(0=no, 1=yes)* J) time to positivity *(TTP: 0=not determined, 1= sample positive in 0-24h, 2= sample positive in 24-48h, 3= sample positive in 48-72h, 4= sample positive in 72-96h, 5= sample positive in 96-120h, 6= sample positive in 120-144h, 7= sample positive in 144-168h, 8= sample positive in 168-192h)* K) no polymicrobial growth *(0=no (other relevant pathogen), 1=yes (only S. saccharolyticus))* L) temperature at admission *(in °C)* M) maximal temperature during stay *(in °C)* N) Body temperature >38° +/- 2 days before/after sampling *(0=no, 1=yes)* O) heart rate *(in beats per minute)* P) blood pressure, systolic *(in mmHg)* Q) blood pressure, diastolic *(in mmHg)* R) C-reactive protein *(in mg/L)* S) C-reactive protein > 80mg/L(*0=no, 1=yes)* T) Leukocyte count *(in 10^9^/L)* U) Procalcitonin *(in ng/mL)* V) medical report indicates infection *(0=no, 1=yes)* W) antibiotic therapy *(0=no, 1=yes)* X) symptom improvement after therapy *(0=no, 1=yes)* Y) improvement labaratory *(0=no, 1=yes)* Z) ICU stay *(0=no, 1=yes)* AA) length of ICU stay *(in days)* AB) NGS *(0= NGS not performed, 1=NGS performed)* AC) CladeAB *(0=Clade A, 1=Clade B)* AD) Infection Score – Points AE) CutOff3: infection likely *(0=infection unlikely, 1=infection likely)* | | | | | | | | | | | | | | | | | | | | | | | | | | | | | | |
| --- | --- | --- | --- | --- | --- | --- | --- | --- | --- | --- | --- | --- | --- | --- | --- | --- | --- | --- | --- | --- | --- | --- | --- | --- | --- | --- | --- | --- | --- | --- |
| A | **B** | **C** | **D** | **E** | **F** | **G** | **H** | **I** | **J** | **K** | **L** | **M** | **N** | **O** | **P** | **Q** | **R** | **S** | **T** | **U** | **V** | **W** | **X** | **Y** | **Z** | **AA** | **AB** | **AC** | **AD** | **AE** |
| 1 | 0 | 54,9 | 1 | 1 | 0 | 16 | 0 | 1 | 4 | 1 | 36,7 | 38,0 | 0 | (-) | 100 | 55 | 149,8 | 1 | 20,0 | (-) | 0 | 1 | 1 | 1 | 1 | 3 | 1 | 1 | 3 | 1 |
| 2 | 0 | 62,7 | 1 | 1 | 0 | 9 | 1 | 1 | 4 | 1 | 37,2 | (-) | 0 | (-) | 140 | 75 | 1,8 | 0 | 3,8 | (-) | 0 | 0 | (-) | (-) | 0 | 0 | 1 | 0 | 2 | 0 |
| 3 | 0 | 80,6 | 1 | 1 | 0 | 22 | 0 | 0 | 0 | 1 | 35,5 | 37,6 | 0 | (-) | 110 | 50 | 140,1 | 1 | 22,7 | (-) | 1 | 1 | 1 | 1 | 1 | 21 | 1 | 0 | 4 | 1 |
| 4 | 0 | 59,4 | 1 | 1 | 0 | 17 | 0 | 0 | 6 | 1 | 36,6 | 38,6 | 0 | 90 | 140 | 85 | 3,5 | 0 | 6,1 | (-) | 0 | 1 | (-) | (-) | 0 | 0 | 1 | 0 | 1 | 0 |
| 5 | 0 | 6,3 | 1 | 1 | 0 | 7 | 0 | 1 | 3 | 1 | (-) | (-) | (-) | (-) | (-) | (-) | 226,2 | 1 | 34,9 | (-) | (-) | (-) | (-) | (-) | (-) | (-) | 1 | 1 | 2 | 0 |
| 6 | 0 | 81,3 | 1 | 1 | 1 | 34 | 1 | 1 | 7 | 1 | 35,0 | 39,1 | 1 | 85 | 145 | 80 | 169,5 | 1 | 9,1 | 1,72 | 1 | 1 | 0 | 0 | 1 | 34 | 1 | 1 | 5 | 1 |
| 7 | 0 | 76,8 | 1 | 1 | 0 | 41 | 0 | 0 | 0 | 1 | 36,8 | 38,2 | 0 | 78 | 110 | 45 | 86,2 | 1 | 12,1 | (-) | 1 | 1 | 1 | 0 | 1 | 20 | 1 | 0 | 4 | 1 |
| 8 | 0 | 60,5 | 1 | 1 | 0 | 13 | 0 | 0 | 3 | 1 | 36,5 | 38,5 | 1 | 70 | 125 | 70 | 41,1 | 0 | 15,7 | (-) | 1 | 1 | 1 | 0 | 0 | 0 | 1 | 0 | 4 | 1 |
| 9 | 0 | 83,1 | 1 | 1 | 0 | 15 | 0 | 1 | 3 | 1 | 36,9 | 37,5 | 0 | 85 | 125 | 65 | 4,8 | 0 | 3,5 | (-) | 0 | 0 | (-) | 1 | 0 | 0 | 1 | 1 | 1 | 0 |
| 10 | 0 | 42,2 | 1 | 1 | 0 | 5 | 0 | 1 | 3 | 1 | (-) | (-) | 0 | 100 | 120 | 90 | 13,8 | 0 | 4,6 | (-) | 1 | 0 | (-) | (-) | 0 | 0 | 1 | 1 | 2 | 0 |
| 11 | 0 | 71,3 | 1 | 1 | 0 | 29 | 0 | 1 | 3 | 0 | 36,5 | 39,5 | 1 | (-) | 125 | 75 | 62,3 | 0 | 7,7 | (-) | 1 | 1 | 1 | 1 | 1 | 13 | 1 | 1 | 3 | 1 |
| 12 | 0 | 53,0 | 1 | 1 | 1 | 1 | 0 | 0 | 5 | 0 | 38,4 | 42,0 | 1 | 90 | 110 | 75 | 9,4 | 0 | 13,8 | (-) | 0 | 0 | (-) | (-) | 1 | 1 | 0 | (-) | 1 | 0 |
| 13 | 0 | 62,1 | 0 | 1 | 0 | 66 | 0 | 1 | 0 | 0 | (-) | 37,5 | (-) | (-) | (-) | (-) | 200,8 | 1 | 7,8 | (-) | (-) | 1 | 0 | 0 | 1 | 3 | 0 | (-) | 1 | 0 |
| 14 | 0 | 46,6 | 1 | 1 | 0 | 38 | 0 | 0 | 7 | 1 | 35,5 | 39,5 | 1 | 70 | 125 | 50 | 340,0 | 1 | 13,5 | (-) | 1 | 1 | 1 | 1 | 1 | 32 | 0 | (-) | 5 | 1 |
| 15 | 0 | 44,2 | 1 | 1 | 0 | 13 | 0 | 1 | 6 | 1 | 35,2 | 40,0 | 1 | (-) | 130 | 65 | 2,4 | 0 | 9,5 | (-) | 1 | 1 | 1 | 1 | 1 | 8 | 0 | (-) | 4 | 1 |
| 16 | 0 | 49,5 | 1 | 1 | 0 | 18 | 0 | 1 | 6 | 0 | 37,0 | 39,0 | 1 | 80 | 110 | 75 | 164,0 | 1 | 9,3 | 42,70 | 1 | 1 | 1 | 1 | 1 | 6 | 0 | (-) | 4 | 1 |
| 17 | 0 | 44,7 | 1 | 1 | 0 | 10 | 0 | 0 | 7 | 1 | 36,1 | 36,9 | 0 | 85 | 135 | 70 | 103,3 | 1 | 10,8 | (-) | 0 | 1 | (-) | 1 | 0 | 0 | 0 | (-) | 2 | 0 |
| 18 | 0 | 54,5 | 1 | 1 | 0 | 33 | 0 | 0 | 8 | 1 | 37,0 | 39,5 | 1 | 140 | 140 | 70 | 173,1 | 1 | 18,6 | 0,20 | 1 | 1 | 1 | 1 | 1 | 15 | 0 | (-) | 5 | 1 |
| 19 | 0 | 29,5 | 1 | 1 | 0 | 49 | 0 | 1 | 7 | 1 | 36,4 | 38,7 | 1 | 100 | 135 | 85 | 138,9 | 1 | 16,6 | 1,00 | 1 | 1 | 0 | 0 | 1 | 49 | 0 | (-) | 4 | 1 |
| 20 | 0 | 23,2 | 1 | 0 | 0 | 0 | 0 | 0 | 5 | 1 | 38,8 | 38,8 | 1 | 80 | 120 | 80 | 7,0 | 0 | 13,8 | (-) | 1 | 0 | 0 | (-) | 0 | 0 | 0 | (-) | 3 | 1 |
| 21 | 0 | 72,9 | 0 | 1 | 0 | 25 | 0 | 1 | 0 | 1 | 36,0 | 39,1 | 1 | 90 | 150 | 85 | 3,2 | 0 | 5,4 | (-) | 1 | 1 | 1 | 1 | 1 | 5 | 0 | (-) | 4 | 1 |
| 22 | 0 | 23,2 | 0 | 0 | 0 | 5 | 0 | 0 | 0 | 0 | (-) | 37,3 | 0 | (-) | 140 | 80 | 104,7 | 1 | 13,6 | (-) | (-) | 1 | 1 | (-) | 0 | 0 | 0 | (-) | 2 | 0 |
| 23 | 0 | 56,2 | 0 | 1 | 0 | 5 | 0 | 1 | 0 | 1 | 38,2 | 38,2 | 1 | (-) | 130 | 90 | 5,8 | 0 | 9,3 | (-) | 0 | 1 | 1 | 1 | (-) | (-) | 0 | (-) | 3 | 1 |
| 24 | 1 | 28,8 | 1 | 0 | 0 | 32 | 0 | 0 | 7 | 1 | 36,5 | 37,9 | 0 | 90 | 140 | 80 | 96,5 | 1 | 16,3 | (-) | 1 | 1 | 1 | 1 | 1 | 14 | 0 | (-) | 4 | 1 |
| 25 | 0 | 46,1 | 0 | 1 | 0 | 23 | 0 | 1 | 0 | 1 | 36,6 | 40,0 | 0 | (-) | 125 | 65 | 35,0 | 0 | 15,9 | (-) | 1 | 1 | 1 | 1 | 1 | 12 | 0 | (-) | 3 | 1 |
| 26 | 0 | 55,8 | 0 | 1 | 0 | 13 | 0 | 1 | 0 | 1 | 36,0 | 38,0 | 0 | 90 | 120 | 60 | (-) | (-) | 19,4 | (-) | 0 | 1 | (-) | (-) | 0 | 0 | 0 | (-) | 1 | 0 |
| 27 | 0 | 29,0 | 1 | 1 | 0 | 4 | 0 | 0 | 6 | 1 | 39,0 | 40,1 | 1 | 99 | 151 | 82 | 86,9 | 1 | 14,2 | (-) | 1 | 1 | 1 | 1 | 0 | 0 | 0 | (-) | 5 | 1 |
| 28 | 0 | 69,9 | 0 | 1 | 0 | 12 | 0 | 1 | 0 | 1 | 37,0 | 37,7 | 0 | 80 | 150 | 75 | 259,8 | 1 | 10,4 | (-) | 0 | 1 | (-) | 1 | (-) | (-) | 0 | (-) | 2 | 0 |
| 29 | 1 | 85,9 | 0 | 1 | 0 | 35 | 0 | 1 | 0 | 1 | 36,5 | (-) | (-) | (-) | (-) | (-) | 146,7 | 1 | 7,1 | (-) | 1 | 1 | 0 | 0 | 1 | 9 | 0 | (-) | 3 | 1 |
| 30 | 0 | 51,5 | 1 | 1 | 0 | 14 | 0 | 0 | 6 | 0 | 36,5 | 37,2 | 0 | 94 | 135 | 85 | 59,4 | 0 | 10,6 | 3,40 | 0 | 1 | 1 | 1 | 1 | 3 | 0 | (-) | 1 | 0 |
| 31 | 0 | 74,4 | 0 | 1 | 0 | 19 | 0 | 0 | 0 | 1 | 35,7 | 37,5 | 0 | 75 | 130 | 70 | 107,7 | 1 | 8,8 | (-) | 0 | 0 | (-) | (-) | 0 | 0 | 0 | (-) | 2 | 0 |
| 32 | 0 | 63,9 | 0 | 1 | 0 | 21 | 0 | 1 | 0 | 0 | 36,5 | 37,0 | 0 | 90 | 103 | 68 | 140,0 | 1 | 10,4 | (-) | 0 | 0 | (-) | (-) | 0 | 0 | 0 | (-) | 1 | 0 |
| 33 | 0 | 60,1 | 1 | 1 | 0 | 43 | 0 | 1 | 6 | 1 | 34,5 | 38,9 | 1 | 100 | 130 | 50 | 254,1 | 1 | 11,3 | 0,90 | 1 | 1 | 1 | 1 | 1 | 43 | 1 | 0 | 5 | 1 |
| 34 | 0 | 33,0 | 0 | 1 | 0 | (-) | 0 | (-) | 0 | 1 | (-) | (-) | (-) | (-) | (-) | (-) | (-) | (-) | (-) | (-) | (-) | (-) | (-) | (-) | (-) | (-) | 0 | (-) | 1 | 0 |
| 35 | 0 | 44,3 | 1 | 1 | 0 | 49 | 0 | 1 | 5 | 1 | 38,5 | 42,0 | 1 | 70 | 92 | 50 | 240,1 | 1 | 6,0 | 0,40 | 1 | 1 | 0 | 0 | 1 | 49 | 0 | (-) | 4 | 1 |
| 36 | 0 | 64,2 | 1 | 0 | 0 | 81 | 0 | 1 | 6 | 1 | 35,8 | 39,1 | 1 | 100 | 150 | 70 | 263,2 | 1 | 13,5 | (-) | 1 | 1 | (-) | 1 | 1 | 2 | 0 | (-) | 4 | 1 |
| 37 | 0 | 59,2 | 0 | 1 | 0 | (-) | 0 | (-) | 0 | 1 | (-) | 0,0 | (-) | (-) | (-) | (-) | (-) | (-) | (-) | (-) | (-) | (-) | (-) | (-) | (-) | (-) | 0 | (-) | 1 | 0 |
| 38 | 1 | 16,4 | 0 | 0 | 0 | 11 | 0 | 0 | 0 | 0 | 37,2 | 39,0 | 0 | 97 | 120 | 71 | 67,2 | 0 | 8,2 | (-) | 1 | 1 | 1 | 1 | 0 | 0 | 0 | (-) | 2 | 0 |
| 39 | 0 | 53,5 | 0 | 1 | 0 | 0 | 0 | 1 | 0 | 1 | (-) | (-) | (-) | (-) | 110 | 70 | 1,9 | 0 | 6,7 | (-) | 0 | 1 | (-) | (-) | 0 | 0 | 0 | (-) | 1 | 0 |
| 40 | 0 | 69,8 | 1 | 1 | 0 | 72 | 0 | 1 | 6 | 1 | (-) | 40,0 | 1 | 91 | 124 | 71 | 253,4 | 1 | 10,2 | 0,33 | 1 | 1 | 0 | 0 | 0 | 0 | 1 | 0 | 4 | 1 |
| 41 | 0 | 40,8 | 1 | 1 | 0 | 3 | 0 | 0 | 8 | 1 | 37,7 | 37,7 | 0 | 112 | 144 | 80 | 265,5 | 1 | 13,3 | (-) | 1 | 1 | 1 | 1 | 0 | 0 | 0 | (-) | 4 | 1 |
| 42 | 0 | 40,7 | 1 | 1 | 0 | 41 | 1 | 1 | 5 | 1 | (-) | 39,0 | 1 | 80 | 140 | 70 | 36,5 | 0 | 10,6 | (-) | 1 | 1 | (-) | (-) | 1 | 39 | 0 | (-) | 4 | 1 |
| 43 | 0 | 57,0 | 1 | 1 | 0 | 62 | 0 | 1 | 6 | 0 | (-) | 39,5 | 1 | 104 | 100 | 48 | 99,4 | 1 | 9,1 | 0,63 | 1 | 1 | 1 | (-) | 1 | 46 | 0 | (-) | 4 | 1 |
| 44 | 0 | 49,4 | 1 | 0 | 1 | 9 | 1 | 0 | 6 | 0 | 34,0 | 40,0 | 1 | 95 | 125 | 70 | 219,8 | 1 | 4,4 | 0,16 | 1 | 1 | 0 | 0 | 1 | 9 | 0 | (-) | 4 | 1 |
| 45 | 0 | 65,8 | 1 | 1 | 0 | 60 | 1 | 1 | 5 | 0 | 36,9 | 38,8 | 0 | 112 | 116 | 70 | 42,2 | 0 | 12,4 | 0,22 | 1 | 1 | 1 | 1 | 1 | 55 | 0 | (-) | 3 | 1 |
| 46 | 0 | 74,8 | 1 | 1 | 0 | 12 | 0 | 0 | 5 | 0 | 35,6 | 38,3 | 1 | 70 | 110 | 50 | 42,5 | 0 | 16,5 | (-) | 1 | 1 | 1 | 1 | 1 | 7 | 0 | (-) | 3 | 1 |
| 47 | 0 | 79,7 | 1 | 1 | 0 | 8 | 0 | 1 | 6 | 1 | 34,7 | 39,1 | 1 | 100 | 90 | 50 | 310,7 | 1 | 8,6 | 1,24 | 1 | 1 | (-) | 1 | 1 | 7 | 0 | (-) | 4 | 1 |
| 48 | 0 | 69,1 | 0 | 1 | 0 | 14 | 0 | 0 | 0 | 0 | 37,6 | 37,7 | 0 | 90 | 200 | 84 | 282,5 | 1 | 5,3 | (-) | (-) | 0 | (-) | (-) | 1 | 1 | 0 | (-) | 1 | 0 |
| 49 | 0 | 45,6 | 1 | 1 | 0 | 17 | 0 | 1 | 5 | 1 | 38,5 | 40,2 | 1 | 90 | 112 | 54 | 96,7 | 1 | 0,7 | 0,58 | 1 | 1 | (-) | (-) | 0 | 0 | 1 | 1 | 4 | 1 |
| 50 | 0 | 46,6 | 0 | 1 | 0 | 20 | 0 | 1 | 0 | 1 | (-) | 40,0 | 1 | 65 | 140 | 80 | 160,2 | 1 | 11,6 | (-) | 1 | 1 | 1 | 1 | 1 | 1 | 0 | (-) | 5 | 1 |
| 51 | 1 | 46,5 | 0 | 0 | 0 | 10 | 0 | 0 | 0 | 1 | (-) | (-) | (-) | (-) | (-) | (-) | 13,5 | 0 | (-) | (-) | 0 | 1 | (-) | 1 | 0 | 0 | 0 | (-) | 1 | 0 |
| 52 | 0 | 47,2 | 0 | 1 | 0 | 12 | 0 | 1 | 0 | 1 | 37,4 | 38,1 | 1 | 100 | 130 | 70 | 57,5 | 0 | 6,3 | (-) | 0 | 1 | (-) | (-) | 1 | 1 | 0 | (-) | 2 | 0 |
| 53 | 0 | 20,3 | 1 | 0 | 0 | 8 | 0 | 0 | 5 | 0 | 36,0 | 36,9 | 0 | 95 | 153 | 80 | 159,7 | 1 | 6,5 | (-) | 0 | 1 | 1 | 1 | 0 | 0 | 0 | (-) | 2 | 0 |
| 54 | 0 | 61,7 | 0 | 1 | 0 | 2 | 0 | 0 | 0 | 0 | (-) | (-) | 0 | 70 | 135 | 90 | (-) | (-) | (-) | (-) | 0 | 1 | (-) | (-) | 0 | 0 | 0 | (-) | 0 | 0 |
| 55 | 0 | 76,5 | 0 | 1 | 0 | 30 | 0 | 1 | 0 | 1 | 36,0 | 37,5 | 0 | 128 | 200 | 90 | 240,4 | 1 | 9,1 | 4,52 | (-) | 1 | (-) | 1 | 1 | 4 | 0 | (-) | 2 | 0 |
| 56 | 1 | 90,1 | 1 | 1 | 0 | 13 | 0 | 1 | 6 | 0 | 35,5 | 38,5 | 0 | 90 | 190 | 80 | 158,7 | 1 | 7,4 | (-) | 1 | 1 | 0 | 0 | 1 | 13 | 0 | (-) | 2 | 0 |
| 57 | 1 | 68,1 | 1 | 1 | 1 | 34 | 0 | 0 | 5 | 0 | 37,0 | 40,2 | 1 | 140 | 160 | 95 | 352,1 | 1 | 9,8 | 9,11 | 1 | 1 | 0 | 0 | 1 | 30 | 1 | 0 | 3 | 1 |
| 58 | 0 | 26,9 | 1 | (-) | (-) | 1 | 0 | (-) | 4 | 1 | (-) | (-) | (-) | (-) | (-) | (-) | (-) | (-) | (-) | (-) | (-) | (-) | (-) | (-) | (-) | (-) | 0 | (-) | 1 | 0 |
| 59 | 1 | 36,8 | 1 | 1 | 0 | 179 | 0 | 0 | 4 | 1 | (-) | 40,2 | 1 | 140 | 110 | 80 | 144,4 | 1 | 8,3 | (-) | (-) | 1 | (-) | (-) | 0 | 0 | 1 | 0 | 3 | 1 |
| 60 | 0 | 61,3 | 1 | 1 | 0 | 79 | 0 | 0 | 4 | 1 | (-) | 37,0 | (-) | (-) | (-) | (-) | 74,8 | 0 | 13,6 | 1,28 | 1 | 1 | 1 | 1 | 1 | 1 | 0 | (-) | 3 | 1 |
| 61 | 0 | 47,9 | 1 | 1 | 0 | 11 | 0 | 1 | 5 | 1 | 36,0 | 39,2 | 1 | 90 | 125 | 70 | 75,7 | 0 | 6,8 | (-) | 1 | 1 | 1 | 1 | 1 | 1 | 0 | (-) | 4 | 1 |
| 62 | 0 | 39,6 | 1 | 1 | 0 | 7 | 0 | 0 | 4 | 1 | 36,8 | 37,4 | 0 | 65 | 110 | 60 | 76,2 | 0 | 9,3 | (-) | 1 | 0 | (-) | (-) | 1 | 3 | 1 | 0 | 2 | 0 |
| 63 | 1 | 57,1 | 1 | 1 | 0 | 112 | 0 | 1 | 5 | 0 | 33,0 | 37,2 | 0 | 104 | 100 | 40 | 317,9 | 1 | 20,3 | 3,55 | 1 | 1 | (-) | (-) | 1 | 112 | 0 | (-) | 2 | 0 |
| 64 | 0 | 24,7 | 0 | 1 | 0 | (-) | 0 | (-) | 0 | 0 | (-) | (-) | (-) | (-) | (-) | (-) | (-) | (-) | (-) | (-) | (-) | (-) | (-) | (-) | 0 | 0 | 0 | (-) | 0 | 0 |
| 65 | 0 | 58,4 | 0 | 0 | 0 | 17 | 1 | 0 | 0 | 1 | (-) | (-) | (-) | (-) | (-) | (-) | 233,7 | 1 | 7,2 | (-) | 1 | 1 | 1 | 1 | 1 | 1 | 0 | (-) | 5 | 1 |
| 66 | 0 | 42,3 | 1 | 1 | 0 | 14 | 0 | 0 | 4 | 1 | (-) | 39,0 | 1 | 78 | 130 | 70 | 140,8 | 1 | 6,6 | (-) | 1 | 1 | 1 | 1 | 1 | (-) | 0 | (-) | 5 | 1 |
| 67 | 0 | 60,1 | 1 | 1 | 0 | 42 | 0 | 0 | 6 | 0 | (-) | 38,7 | 1 | 100 | 104 | 68 | 69,1 | 0 | 16,0 | 0,43 | (-) | 1 | (-) | 1 | 1 | 4 | 0 | (-) | 1 | 0 |
| 68 | 0 | 53,7 | 1 | 1 | 1 | 29 | 0 | 0 | 3 | 1 | 39,0 | 39,0 | 1 | 89 | 97 | 60 | 255,9 | 1 | 38,3 | (-) | 1 | 1 | 0 | 0 | 1 | 4 | 1 | 1 | 4 | 1 |
| 69 | 0 | 67,3 | 0 | 1 | 0 | 7 | 0 | 1 | 0 | 0 | (-) | 36,5 | 0 | 80 | 128 | 44 | 69,2 | 0 | 9,9 | 0,09 | 0 | 1 | (-) | (-) | 1 | 3 | 0 | (-) | 0 | 0 |
| 70 | 0 | 64,5 | 0 | 1 | 0 | 10 | 0 | 1 | 0 | 1 | (-) | (-) | (-) | (-) | (-) | (-) | 32,0 | 0 | 8,8 | (-) | 0 | 1 | (-) | (-) | (-) | (-) | 0 | (-) | 1 | 0 |
| 71 | 0 | 54,7 | 1 | 1 | 0 | 28 | 0 | 1 | 7 | 0 | 36,0 | 37,6 | 0 | 80 | 140 | 50 | 60,9 | 0 | 12,5 | 0,26 | 1 | 1 | 1 | 1 | 1 | 18 | 0 | (-) | 2 | 0 |
| 72 | 0 | 58,7 | 0 | (-) | (-) | (-) | 0 | (-) | 0 | (-) | (-) | (-) | (-) | (-) | (-) | (-) | (-) | (-) | (-) | (-) | (-) | (-) | (-) | (-) | (-) | (-) | 0 | (-) | 0 | 0 |
| 73 | 1 | 57,8 | 1 | 1 | 0 | 12 | 0 | 0 | 3 | 1 | 36,6 | 37,5 | 0 | (-) | (-) | (-) | 171,8 | 1 | 9,7 | (-) | (-) | 1 | (-) | (-) | 1 | 2 | 1 | 1 | 2 | 0 |
| 74 | 0 | 49,8 | 1 | 1 | 0 | 6 | 0 | 1 | 3 | 1 | (-) | 37,7 | 0 | 69 | 94 | 58 | 12,3 | 0 | 16,0 | (-) | 0 | 1 | (-) | (-) | 1 | 1 | 1 | 1 | 1 | 0 |
| 75 | 0 | 35,8 | 0 | 0 | 0 | 4 | 0 | 0 | 0 | 1 | (-) | (-) | (-) | (-) | (-) | (-) | 1,0 | 0 | 7,9 | (-) | (-) | (-) | (-) | (-) | 0 | 0 | 0 | (-) | 1 | 0 |
| 76 | 1 | 62,2 | 1 | 1 | 0 | 41 | 0 | 0 | 3 | 0 | (-) | 39,1 | 1 | 95 | 120 | 50 | 219,0 | 1 | 16,9 | 8,81 | 1 | 1 | 0 | 0 | 1 | 34 | 0 | (-) | 3 | 1 |
| 77 | 0 | 69,4 | 0 | 0 | 0 | 16 | 0 | 0 | 0 | 1 | (-) | (-) | 0 | 90 | 110 | 75 | 47,7 | 0 | 9,9 | (-) | 0 | 1 | (-) | (-) | 1 | 1 | 0 | (-) | 1 | 0 |
| 78 | 0 | 58,7 | 1 | 1 | 0 | 227 | 0 | 0 | 7 | 0 | 37,5 | 40,1 | 1 | 104 | 120 | 68 | 103,6 | 1 | 6,4 | 1,33 | 1 | 1 | 0 | 0 | 1 | 141 | 0 | (-) | 3 | 1 |
| 79 | 0 | 33,3 | 0 | 1 | 0 | 36 | 0 | 0 | 0 | 1 | 37,2 | 37,9 | (-) | (-) | (-) | (-) | 3,4 | 0 | 8,1 | (-) | (-) | 1 | (-) | (-) | 1 | 6 | 0 | (-) | 1 | 0 |
| 80 | 0 | 83,8 | 0 | 1 | 0 | 6 | 0 | 1 | 0 | 1 | 38,0 | 38,0 | 0 | (-) | (-) | (-) | 0,4 | 0 | 8,4 | (-) | 0 | 1 | (-) | (-) | 0 | 0 | 0 | (-) | 1 | 0 |
| 81 | 0 | 71,0 | 0 | 1 | 0 | 38 | 0 | 0 | 4 | 0 | 37,6 | 37,6 | 0 | 110 | 90 | 50 | 161,1 | 1 | 15,5 | (-) | 1 | 1 | 1 | 0 | 1 | 2 | 0 | (-) | 3 | 1 |
| 82 | 1 | 43,3 | 0 | 1 | 0 | 1 | 0 | (-) | 0 | 1 | (-) | (-) | (-) | (-) | (-) | (-) | (-) | (-) | (-) | (-) | (-) | (-) | (-) | (-) | 0 | 0 | 0 | (-) | 1 | 0 |
| 83 | 0 | 78,4 | 1 | 1 | 0 | 37 | 0 | 1 | 4 | 1 | 36,5 | 38,9 | 1 | 100 | 80 | 40 | 385,7 | 1 | 17,5 | (-) | 1 | 1 | 1 | (-) | 1 | 14 | 0 | (-) | 5 | 1 |
| 84 | 0 | 51,6 | 0 | 0 | 0 | 5 | 0 | 0 | 0 | 0 | (-) | (-) | (-) | (-) | (-) | (-) | (-) | (-) | (-) | (-) | 0 | 1 | (-) | (-) | 0 | 0 | 0 | (-) | 0 | 0 |
| 85 | 1 | 54,0 | 0 | 1 | 0 | 3 | 0 | 0 | 0 | 0 | (-) | (-) | (-) | (-) | (-) | (-) | 7,7 | 0 | 6,7 | (-) | (-) | 1 | (-) | (-) | 0 | 0 | 0 | (-) | 0 | 0 |
| 86 | 0 | 66,3 | 1 | 1 | 0 | 4 | 0 | 1 | 3 | 1 | 36,8 | 38,5 | 1 | 105 | 125 | 60 | 20,0 | 0 | 9,7 | (-) | 1 | 1 | 1 | 0 | 0 | 0 | 0 | (-) | 4 | 1 |
| 87 | 0 | 62,8 | 1 | 1 | 0 | 21 | 0 | 0 | 2 | 0 | (-) | (-) | 1 | (-) | (-) | (-) | 205,3 | 1 | 12,1 | (-) | 1 | 1 | 1 | 1 | 0 | 0 | 0 | (-) | 4 | 1 |
| 88 | 0 | 62,8 | 1 | 0 | 0 | 26 | 0 | 0 | 3 | 1 | 36,9 | 37,3 | 0 | 50 | 160 | 68 | 1,4 | 0 | 9,7 | 0,08 | 0 | 1 | (-) | (-) | 1 | 1 | 0 | (-) | 1 | 0 |
| 89 | 0 | 55,7 | 0 | 1 | 0 | 5 | 0 | 0 | 0 | 0 | 36,0 | 37,0 | 0 | 80 | 150 | 80 | (-) | (-) | (-) | (-) | 0 | 0 | (-) | (-) | 0 | 0 | 0 | (-) | 0 | 0 |
| 90 | 0 | 68,9 | 1 | 1 | 0 | 30 | 0 | 1 | 3 | 0 | (-) | (-) | (-) | (-) | (-) | (-) | (-) | (-) | (-) | (-) | (-) | (-) | (-) | (-) | (-) | (-) | 1 | 1 | 0 | 0 |
| 91 | 1 | 21,4 | 0 | 0 | 0 | 0 | 0 | 0 | 0 | (-) | (-) | (-) | (-) | (-) | (-) | (-) | (-) | (-) | (-) | (-) | (-) | (-) | (-) | (-) | (-) | (-) | 0 | (-) | 0 | 0 |
| 92 | 0 | 73,6 | 0 | (-) | 0 | 0 | 0 | (-) | 0 | (-) | (-) | (-) | (-) | (-) | (-) | (-) | (-) | (-) | (-) | (-) | (-) | (-) | (-) | (-) | (-) | (-) | 0 | (-) | 0 | 0 |
| 93 | 0 | 54,2 | 1 | 1 | 0 | 8 | 0 | 0 | 3 | 1 | (-) | (-) | (-) | (-) | (-) | (-) | 64,2 | (-) | 12,7 | (-) | (-) | 1 | (-) | (-) | (-) | (-) | 1 | 1 | 1 | 0 |
