# Supplementary material for: Clinical relevance of Staphylococcus saccharolyticus detection in human samples: a retrospective cohort study
Source: Infection. 2024 Jul 4;53(1):145–53. doi: 10.1007/s15010-024-02334-6 (PMC11825615; doi:10.1007/s15010-024-02334-6)
Supplement: Supplementary file 2 — Supplementary Material 2 [file 15010_2024_2334_MOESM2_ESM.docx]

**Online Supplement to: Michels et al. Clinical relevance of *Staphylococcus saccharolyticus* detection in human samples: a retrospective cohort study**

**Supplementary Table S2.** Data on infection and clinical characteristics per patient and clade

|  | *0=infection unlikely*  *1=infection likely (based on score)* | *Medical report indicates infection (0=no, 1=yes)* | **Type of infection (based on medical report)** |
| --- | --- | --- | --- |
| **Clade A (ID nr.)** |  |  |  |
| **2** | 0 | 0 | Bypass operation. No evidence of infection |
| **3** | 1 | 1 | Sepsis, Suspected pneumonia with radiographic infiltrates |
| **4** | 0 | 0 | Patient under chemotherapy. No evidence of infection |
| **7** | 1 | 1 | pneumonia |
| **8** | 1 | 1 | foreign body related infection (urinary catheter) |
| **33** | 1 | 1 | Soft tissue infection after polytrauma |
| **40** | 1 | 1 | fever without source |
| **57** | 1 | 1 | Sepsis |
| **59** | 1 | - | No clear indication of infection. Repeated fever and elevated markers of inflammation in the context of acute leukaemia |
| **62** | 0 | 1 | myocarditis |
| **Clade B (ID nr.)** |  |  |  |
| **1** | 1 | 0 | Clinical deterioration after aortic valve replacement. No evidence of infection. |
| **5** | 0 | - | medical report missing |
| **6** | 1 | 1 | fever without source |
| **9** | 0 | 0 | Prosthesis change, intraoperative pathogen detection. No evidence of infection postoperatively |
| **10** | 0 | 1 | Redness, swelling, pain at the port entry site |
| **11** | 1 | 1 | Postoperative persistent fever, unclear focus |
| **49** | 1 | 1 | fever without source |
| **68** | 1 | 1 | sepsis |
| **73** | 0 | - | Elevated laboratory parameters, no clinical evidence of infection |
| **74** | 0 | 0 | Uncomplicated postoperative course after heart surgery |
| **90** | 0 | - | medical report missing |
| **93** | 0 | - | medical report missing |
